# A Characterization of Women Living with HIV in Belgium

**DOI:** 10.1155/2024/5590523

**Published:** 2024-09-26

**Authors:** Rakan Nasreddine, Jean Cyr Yombi, Gilles Darcis, Maartje Van Frankenhuijsen, Lida Van Petersen, Chloé Abels, Sofia Dos Santos Mendes, Marc Delforge, Stéphane De Wit

**Affiliations:** ^1^ Saint‐Pierre University Hospital, Brussels, Belgium; ^2^ Cliniques Universitaires Saint-Luc, Brussels, Belgium; ^3^ Liège University Hospital, Liège, Belgium; ^4^ Institute of Tropical Medicine, Antwerp, Belgium; ^5^ MSD, Watermael-Boitsfort, Belgium

## Abstract

**Objectives:**

The primary objective of this study was to characterize women living with HIV (WLWH) in Belgium. The secondary objective was an exploratory analysis comparing women and men living with HIV (MLWH).

**Methods:**

This was a cross-sectional, observational, multicenter study. Inclusion criteria for the primary objective were all adult treatment-naïve and experienced WLWH actively being treated at one of the participating centers. For the secondary objective, inclusion criteria were all adult naïve and experienced women and MLWH, actively being treated at a single participating center. Data were collected between December 2022 and March 2023. A multivariable analysis was performed on all women included to evaluate for associations with having hypertension (HTN) or being virologically nonsuppressed (HIV-1 VL ≥200 copies/mL). In the exploratory analysis of women and MLWH, a multivariable analysis was carried out to evaluate whether female gender was associated with having HTN or being virologically nonsuppressed.

**Results:**

Overall, 2797 WLWH were included. The majority were Black (73.5%) and 48.5% were aged ≥50 years. The most common comorbidity was HTN (17.3%) and most individuals were virologically suppressed (HIV-1 VL <50 copies/mL; 85.6%). Black race was associated with having HTN (*p* < 0.0001). Prior AIDS-defining illness (*p* = 0.02) and a CD4^+^ T-cell count <500 cells/µL (*p* < 0.0001) were associated with being nonsuppressed. A total of 1094 WLWH and 1878 MLWH were included in the exploratory analysis. HTN was higher among WLWH (20.2% vs. 12% MLWH). Female gender was not found to be associated with having HTN (*p* = 0.86) or being nonsuppressed (*p* = 0.14).

**Conclusion:**

In this analysis of WLWH in Belgium, the results depict an ageing population that is predominantly Black. The most common comorbidity observed was HTN. Women had a low rate of virologic nonsuppression, and female gender was not associated with being nonsuppressed.

## 1. Introduction

Of the estimated 39 million people living with HIV (PLWH) worldwide in 2022, women and girls represented 53% of this population and accounted for 46% of all new infections [1]. Typically, physicians tend to apply the same approach to the management of HIV for both women and men, despite the fact that many gender-specific differences exist. These include varying pharmacokinetic and pharmacodynamics profiles, differing immune responses, experiencing gender-related events such as pregnancy and menopause, and socioeconomic factors that can impact health-seeking behavior and access to healthcare, all of which can result in different treatment outcomes between women and men [2, 3]. In addition, women living with HIV (WLWH) are greatly under-represented in many areas of HIV research, and even when they are involved, those studies are often underpowered to provide women-specific evidence [4].

By the end of 2022, women and girls accounted for 34% of all Belgian PLWH under medical care and 32% of all newly diagnosed persons [5]. Many studies have been done on the Belgian HIV population in the past, but a detailed descriptive examination of WLWH, as well as a comparative analysis of women vs. men living with HIV (MLWH), is yet to be performed. As such, understanding the characteristics of WLWH, including newly diagnosed women, and how these may differ from those of MLWH, would greatly contribute to advancing gender-specific HIV care.

## 2. Methods

### 2.1. Study Design and Objectives

This was an observational, cross-sectional, multicenter study. The primary objective was to characterize WLWH in Belgium. The secondary objective was to perform an exploratory analysis comparing WLWH and MLWH.

### 2.2. Study Population and Variables

Inclusion criteria for the primary objective were treatment-naïve and experienced cis and transgender WLWH, aged 18 years and above, actively being treated at one of the participating centers. This was defined as having had at least 1 consultation with their HIV specialist (either in-person, by telephone, or by videoconferencing) within 12 months prior to June 1, 2022. For the secondary objective, inclusion criteria were naïve and experienced cis and transgender WLWH and MLWH, aged 18 years and above, actively being treated at a single participating center, defined as having had at least 1 consultation with their HIV specialist (either in-person, by telephone, or by videoconferencing) within 12 months prior to June 1, 2022. There were no exclusion criteria.

The study variables included in this study were captured electronically from routine clinical practice and archived at each participating center. The data were then collected by each center between December 2022 and March 2023, and these included age, gender, race, body mass index (BMI), comorbidities, time since HIV diagnosis, method of acquisition of HIV, previous AIDS-defining conditions, treatment status (naïve, experienced <6 months, and experienced ≥6 months), time since initiation of first antiretroviral therapy (ART) and number of regimens prior to current treatment, current ART regimen (indicated by class), CD4^+^ T-cell count nadir, current CD4^+^ and CD8^+^ T-cell counts, current HIV-1 viral load (VL), estimated glomerular filtration rate (eGFR), total cholesterol (TC), triglycerides, low-density lipoprotein (LDL), high-density lipoprotein (HDL), and fasting plasma glucose (FPG). If more than one result was available for a specific variable to be collected, then the most recent value prior to June 1, 2022 was utilized.

### 2.3. Data Source and Ethics Considerations

Data were collected from four designated HIV reference centers (HRCs) in Belgium. These HRCs are tertiary care centers that have the largest proportion of WLWH in follow-up. The significantly large number of participants included in this analysis, representing 45.2% of the overall female HIV population in Belgium [6], along with the geographical distribution of the participating HRCs, provided not only an ample sampling of WLWH in Belgium, but also ensured an adequate evaluation of gender-specific characteristics.

This study was conducted according to Good Pharmacoepidemiology Practice guidelines and local regulations. Ethical approval was obtained from all participating centers prior to the commencement of the study. Informed consent requirement was waived because this was a cross-sectional study of retrospective variables, and each study site ensured that the dataset for each participant underwent deidentification prior to collection.

### 2.4. Statistical Analysis

Characteristics of the study population were depicted using descriptive statistics. Categorical data were reported as the frequency and percentage, while continuous data were conveyed as the median and interquartile range (IQR). Pairwise deletion was applied when treating missing data. For the primary objective, results were portrayed for the overall population of WLWH and for the subgroups of women diagnosed within the last two years and transgender women. Furthermore, a multivariable analysis was performed on all WLWH in order to evaluate for associations with the following outcomes: having hypertension (HTN; the most frequently observed comorbidity) or being virologically nonsuppressed (HIV-1 VL ≥200 copies/mL). For the exploratory analysis of WLWH and MLWH being treated at one of the participating centers, a multivariable analysis was performed to evaluate whether female or male gender was independently associated with having HTN or being nonsuppressed. All multivariable analyses employed the variable selection technique of stepwise logistic regression. *P* values were two-tailed, and <0.05 was used for statistical significance. All analyses were performed using SAS software v9.4 (SAS Institute Inc., Cary, North Carolina, USA).

## 3. Results

### 3.1. Primary Objective

Overall, 2797 WLWH met the inclusion criteria for the primary objective, of which 97 (3.4%) were diagnosed within the last two years and 51 (2%) were transgender (Table 1). The majority were Black (73.5%) and individuals aged ≥50 years represented 48.5% of the study population. Median (IQR) BMI was 28.3 (24.7–32) kg, while the most common comorbidity was HTN (17.3%). Less frequently observed comorbidities included diabetes mellitus (DM; 7.4%) and non-AIDS-defining malignancies (NADM; 3.8%). The primary mode of HIV acquisition was via heterosexual relations (86%), and the majority of individuals were treatment-experienced (99%). The median (IQR) time on ART was 11.6 (6.4–20.1) years, and the median (IQR) number of ART regimens was 4 (2–7). The most common current ART regimen was a three-drug regimen (3DR) of 2 nucleoside/nucleotide reverse transcriptase inhibitors (NRTIs) + 1 integrase strand transfer inhibitor (INSTI; 34.1%), while a standard two-drug regimen (2DR; INSTI + NRTI or NNRTI) was prescribed in 18.6% of women. Most individuals were virologically suppressed (HIV-1 VL <50 copies/mL; 85.6%), and 72.1% of participants had a CD4^+^ T-cell count ≥500 cells/*µ*L. Overall, the median (IQR) values for eGFR and FPG were 90 (74–106.8) mL/min/1.73 m^2^ and 91 (84–101) mg/dL, respectively (Table 2). LDL and TC were mildly elevated (114 [92–141] mg/dL and 197 [172–226] mg/dL, respectively).

Multivariable regression analysis revealed Black race to be the only significant association with having HTN (odds ratio [OR] 1.99; 95% confidence interval [CI] 1.48–2.68, *p* < 0.0001). Prior AIDS-defining illness (OR 1.67; 95% CI 1.07–2.60, *p*=0.02) and a CD4^+^ T-cell count <500 cells/*µ*L (OR 4.26; 95% CI 2.9–6.25, *p* < 0.0001) were found to be significantly associated with being virologically nonsuppressed (HIV-1 VL ≥200 copies/mL).

### 3.2. Exploratory Analysis

A total of 1094 WLWH and 1878 MLWH were included in the single-center analysis (Table 3). The two groups shared certain similarities such as age ≥50 years (51.4% WLWH vs. 51% MLWH), CD4^+^ T-cell count ≥500 cells/*µ*L (74.8% WLWH vs. 74.3% MLWH), and virologic suppression (88.2% WLWH vs. 88.7% MLWH), whereas they differed in aspects such as the proportion of individuals of Black race (78.7% WLWH vs. 26.1% MLWH) and median CD4^+^ T-cell count nadir (238 cells/µL WLWH vs. 303 cells/*µ*L MLWH). In terms of comorbidities, both groups had similar proportions of individuals with DM (7.3% WLWH vs. 6.5% MLWH), whereas HTN was observed to be higher among WLWH (20.2% vs. 12% MLWH) and ischemic cardiac disease to be slightly higher among MLWH (2.9% vs. 0.8% WLWH). On multivariable analysis, female gender was not found to be independently associated with having HTN (*p*=0.86) or being virologically nonsuppressed (*p*=0.14) nor was male gender (HTN *p*=0.61; virologic nonsuppression *p*=0.26). Black race was again found to be the only significant association with having HTN (OR 2.54; 95% CI 1.98–3.27, *p* < 0.0001), whereas no significant associations with being virologically nonsuppressed were observed.

## 4. Discussion

Despite an increase in the number of women being included in HIV research in recent years, the proportion of women in clinical trials remains relatively low with a median of 19.2% [4]. This study, the first of its kind in Belgium, included 2797 WLWH. Individuals aged ≥50 years represented 48.5% of the cohort, indicative of an ageing HIV population. Indeed, the average age of WLWH in Belgium has steadily increased from 38 years in 2006 to 48 years in 2022 [5]. This increase in age is due, in part, to the improvement in the life expectancy of PLWH as a result of the introduction of more effective antiretroviral therapies, and to the evolution in the care of these individuals. The majority of the women in this study were Black (73.5%), and despite a steady decrease in the number of newly diagnosed Black women in Belgium in recent years (59% decrease in 2021 compared to 2012), this subgroup continues to account for the majority of newly diagnosed women in Belgium, demonstrated by the fact that 53.6% of the women diagnosed within the last two years in our cohort were Black, double that of White women (26.8%). This finding, along with previous reports on the disproportionate impact of HIV on Black women [7], indicates that effective prevention and treatment are not adequately reaching the people who could benefit from them the most. Moreover, when compared to Black WLWH born in Western countries, African-born Black WLWH tend to be diagnosed at a later stage of infection and have less access to optimal HIV care [8, 9]. As such, it is important to note that a sizeable proportion of the Black female HIV population in Belgium were African-born [5]; however, the lack of available data, concerning the place of birth of the participants included in our cohort, precludes us from reporting such data.

It has been suggested that PLWH have higher rates of age-associated nonAIDS-related comorbidities, such as HTN, DM, and ischemic cardiac disease, as compared to seronegative individuals, and that these illnesses may manifest up to 10 years earlier in PLWH [10–12]. Consequently, these so-called “co-morbidities of ageing” are now being managed alongside HIV. Among the women included in this analysis, the most frequently observed comorbidity was HTN (17.3%) and Black race was found to be the only significant association with having HTN (OR 1.99; *p* < 0.0001). In the single-center analysis of women and MLWH, HTN was observed to be higher among WLWH (20.2% vs. 12% MLWH), which is in contrast to the prevalence of HTN in the general population in Belgium, where the male-to-female ratio is 1.3 [13]. Some studies have reported HTN to be higher among WLWH [6], while others have described HTN to be higher among MLWH [14]. In our cohort of women and MLWH, regression analysis revealed neither gender to be significantly associated with having HTN; however, Black race was once again found to be associated with having HTN (OR 2.54; *p* < 0.0001). Indeed, it is known that HTN is more prevalent among Black adults [15]. Factors associated with hypertension among Black individuals are mainly related to unfavorable social determinants of health, such as lower socioeconomic and educational status, and to culturally influenced dietary habits (ingestion of a high-sodium diet) [16]. It is important to note, however, that given Black women represented 73.5% of the overall study population and 78.7% of the women in the single-center analysis, finding Black race to be associated with having HTN in the aforementioned analyses could simply be a consequence of the predominance of Black individuals in our cohort. Overall, DM (7.4%) was the second most common comorbidity observed. In the single-center analysis, WLWH (7.3%) and MLWH (6.5%) had similar proportions of DM, while MLWH were found to have a higher proportion of individuals with ischemic cardiac disease (2.9% vs. 0.8% WLWH). Similar to HTN, there are conflicting reports as to whether WLWH have higher rates of DM and cardiac disease [6, 17, 18] or if MLWH have higher rates of these illnesses [14, 19]. The overall median eGFR observed in our study was within a normal range which is in contrast to some studies reporting a higher incidence of chronic kidney disease in WLWH [20–22]. Median TC and LDL levels were slightly above normal, a finding previously described [23]. The variations in results between our cohort and the aforementioned reports can be most likely explained by differences in the characteristics of the population studied (age, BMI, etc.), duration of follow-up (cross sectional vs. longitudinal), and HIV and non-HIV-related factors such as current/prior abacavir use and current/prior cigarette smoking.

The most common current regimen overall was that of 2 NRTIs + 1 INSTI (34.1%). For the last several years, guidelines for the management of PLWH have recommended the combination of 2 NRTIs and 1 INSTI as a first-line regimen for both naïve and experienced PLWH, and as such, clinicians are utilizing this regimen more and more. This is evidenced by the fact that 49.5% of the women diagnosed within the last 2 years in our study were receiving 2 NRTIs + 1 INSTI. Women receiving standard 2DRs (INSTI + NRTI or NNRTI) represented 18.6% of the overall study population, and this proportion was lower among WLWH (10.6%), when compared to MLWH (17.3%), in the single-center analysis. We hypothesize that the lower prescription of these 2DRs in WLWH, when compared to MLWH, could be due to two factors. First, clinicians may be hesitant to prescribe these treatments to women of childbearing potential as these regimens are not yet approved for use in pregnancy or in women intending to become pregnant. Second, since it has been previously reported that women have lower adherence to ART [24], this in turn could result in some physicians being more inclined to prescribe a 3DR rather than a 2DR.

Overall, 142 (5.1%) women were virologically nonsuppressed. Multivariable analysis showed that having had a prior AIDS-defining illness (OR 1.67; *p*=0.02) or having a CD4^+^ T-cell count <500 cells/*µ*L (OR 4.26; *p* < 0.0001) were significantly associated with being virologically nonsuppressed. The latter finding could simply be a consequence of the progression of illness in these individuals. However, it has been previously reported that viral rebound was more frequently observed among individuals with lower CD4^+^ counts [25]. In the single-center analysis, we observed a slightly higher rate of virologic nonsuppression among WLWH (5%) as compared to MLWH (3.9%). It has been previously reported that women may be at an increased risk of virologic nonsuppression due to higher rates of intolerance to ART or lower adherence to treatment [24]. However, neither gender was found to be independently associated with being virologically nonsuppressed in our study. Overall, 72.1% of women had a CD4^+^ T-cell count >500 cells/*µ*L. Interestingly, the single-center analysis revealed WLWH (74.8%) and MLWH (74.3%) to have similar proportions of individuals with a CD4^+^ T-cell count >500 cells/*µ*L despite WLWH having a noticeably lower median CD4^+^ T-cell count nadir (238 cells/*µ*L vs. 303 cells/*µ*L MLWH). Moreover, WLWH were observed to have a slightly higher CD4^+^/CD8^+^ ratio as well (1 vs. 0.9 MLWH). Indeed, previous studies have reported female gender to be an independent predictor of higher immune reconstitution and CD4^+^/CD8^+^ ratio [20, 26, 27].

This study has some limitations mainly due to its design. Some cardiovascular risk factors such as prior abacavir use and cigarette smoking were not captured. Furthermore, it was not possible to perform a medical file review for each participant to evaluate their adherence to ART and describe their comorbidities, coinfections, and nonantiretroviral medications. This study's strength, however, is the extent of data collected from a very large representative cohort of WLWH in Belgium.

## 5. Conclusion

In this analysis of WLWH in Belgium, the results depict a population that is predominantly Black, including among women diagnosed within the last two years, and approximately half of all women were above the age of 50. The most common comorbidity was HTN and when compared to their male counterparts, WLWH had a slightly higher proportion of individuals with HTN, similar proportion of DM, and lower proportion of ischemic cardiac disease. Most were being treated with a 3DR, with a lower proportion of women receiving a 2DR compared to MLWH. Overall, WLWH had a low rate of virologic nonsuppression, which was slightly higher than that of MLWH, but female gender was not found to be independently associated with being nonsuppressed. Our understanding of how women age with HIV is evolving, and while some differences in the management of WLWH, compared to MLWH already exist, further research is needed to better appreciate the specific needs of WLWH.

## Figures and Tables

**Table 1:** Characteristics of women living with HIV included in this study.

	Overall (*N* = 2797)	Diagnosed ≤2 years (*N* = 97)	Transgender (*N* = 51)
*Age (years)*			
Median (IQR)	49 (41–57)	38 (31–44)	41 (31–51)

*Age (years), n (%)*			
≥50	1356 (48.5)	14 (14.4)	17 (33.3)
<50	1441 (51.5)	83 (85.6)	34 (66.7)

*Race, n (%)*			
Black	2056 (73.5)	52 (53.6)	2 (3.9)
White	565 (20.2)	26 (26.8)	22 (43.1)
Other	115 (4.1)	10 (10.3)	9 (17.7)
Data not available	61 (2.2)	9 (9.3)	18 (35.3)

*Body mass index*			
Median (IQR)	28.3 (24.7–32)	27.8 (24.1–32.1)	25.9 (23.1–30.9)
Data not available, *n* (%)	433 (15.5)	36 (37.1)	16 (31.4)

*Most common comorbidities, n (%)*			
Hypertension	484 (17.3)	5 (5.2)	3 (5.9)
Diabetes mellitus	206 (7.4)	4 (4.1)	1 (2)
Non-AIDS-defining malignancy	106 (3.8)	0 (0)	0 (0)
Neurological disease	33 (1.2)	1 (1)	0 (0)
Ischemic cardiac disease	19 (0.7)	0 (0)	0 (0)

*Method of HIV acquisition, n (%)*			
Heterosexual	2404 (86)	77 (79.4)	3 (5.9)
Vertical transmission	89 (3.2)	1 (1)	0 (0)
Transfusion	75 (2.7)	1 (1)	0 (0)
Homosexual/bisexual	60 (2.1)	8 (8.3)	41 (80.4)
Intravenous drug use	29 (1)	0 (0)	0 (0)
Other	4 (0.1)	0 (0)	0 (0)
Data not available	136 (4.9)	10 (10.3)	7 (13.7)

*Time since HIV diagnosis (years)*			
Median (IQR)	15.6 (9.5–21.2)	0.8 (0.3–1.2)	3.5 (1.1–14.3)
Data not available, *n* (%)	1274 (45.5)	0 (0)	33 (64.7)

*Prior AIDS defining illness*			
*N* (%)	446 (15.9)	8 (8.3)	7 (13.7)
Data not available, *n* (%)	123 (4.4)	9 (9.3)	1 (2)

*NadirCD*4^+^*T-cell count (cells/µL)*			
Median (IQR)	236 (124–376)	375 (228–587)	461 (254–648)
Data not available, *n* (%)	11 (0.4)	2 (2.1)	1 (2)

*HIV treatment status, n (%)*			
Experienced, <6 months	44 (1.6)	34 (35.1)	16 (31.4)
Experienced, ≥6 months	2721 (97.3)	59 (60.8)	34 (66.6)
Experienced, timing not available	3 (0.1)	0 (0)	0 (0)
Naïve	29 (1)	4 (4.1)	1 (2)

*Time on ART (years)*			
Median (IQR)	11.6 (6.4–20.1)	0.9 (0.4–1.5)	3.1 (1–10.6)
Data not available, *n* (%)	38 (1.4)	14 (14.4)	33 (64.7)

*Number of ART regimens received*			
Median (IQR)	4 (2–7)	1 (1–1)	2 (1–2)

*Most common current ART regimen, n (%)*			
2 NRTIs + 1 INSTI	954 (34.1)	48 (49.5)	19 (37.3)
2 NRTIs + 1 NNRTI	696 (24.9)	15 (15.5)	13 (25.5)
1 INSTI + 1 NRTI	405 (14.5)	15 (15.5)	9 (17.6)
2 NRTIs + 1 PI	130 (4.6)	6 (6.2)	5 (9.8)
1 INSTI + 1 NNRTI	116 (4.1)	1 (1)	1 (2)

*CurrentCD*4^+^*T-cell count (cells/µL), n (%)*			
<200	73 (2.6)	8 (8.3)	0 (0)
200–349	191 (6.8)	18 (18.6)	2 (3.9)
350–499	370 (13.2)	12 (12.4)	4 (7.8)
≥500	2016 (72.1)	56 (57.7)	40 (78.4)
Data not available	147 (5.3)	3 (3.1)	5 (9.8)

*CurrentCD*4^+^/*CD*8^+^*ratio*			
Median (IQR)	1 (0.7–1.4)	0.6 (0.4–1.2)	0.9 (0.7–1.4)
Data not available	1122 (40.1)	37 (38.1)	31 (60.8)

*Current HIV-1 viral load (copies/mL), n (%)*			
<50	2393 (85.6)	75 (77.3)	44 (86.3)
50–199	115 (4.1)	3 (3.1)	1 (2)
≥200	142 (5.1)	16 (16.5)	1 (2)
Data not available	147 (5.3)	3 (3.1)	5 (9.8)

IQR, interquartile range; ART, antiretroviral therapy; NRTI, nucleoside/nucleotide reverse transcriptase inhibitor; INSTI, integrase strand transfer inhibitor; NNRTI, non‐nucleoside reverse transcriptase inhibitor; PI, protease inhibitor.

**Table 2:** Laboratory parameters of women living with HIV included in this study.

	Overall		Diagnosed ≤2 years		Transgender	
	*N*	Median (IQR)	*N*	Median (IQR)	*N*	Median (IQR)
Estimated glomerular filtration rate (mL/min/1.73 m^2^)	884	90 (74–106.8)	30	103 (75–114)	9	90 (77–97.5)
Fasting plasma glucose (mg/dL)	1389	91 (84–101)	53	90 (84–97)	17	88 (85–95)
High-density lipoprotein (mg/dL)	1329	59 (49–72)	50	52.5 (44–63)	12	46.9 (35.6–61.5)
Low-density lipoprotein (mg/dL)	1168	114 (92–141)	47	106.4 (84–132)	11	115 (102–139.2)
Triglycerides (mg/dL)	1349	89 (65–126)	50	86.5 (63–138)	12	98 (56.5–159.5)
Total cholesterol (mg/dL)	1369	197 (172–226)	51	183 (154–210)	12	187 (170–210.5)

*N*, number of participants with available data; IQR, interquartile range.

**Table 3:** Baseline characteristics of the participants included in the single-center exploratory analysis.

	Women (*N* = 1094)	Men (*N* = 1878)
*Age (years)*		
Median (IQR)	50 (41–58)	50 (40–59)

*Age (years), n (%)*		
≥50	562 (51.4)	958 (51)
<50	532 (48.6)	920 (49)

*Race, n (%)*		
Black	861 (78.7)	491 (26.1)
White	185 (16.9)	1205 (64.2)
Other	21 (1.9)	62 (3.3)
Data not available	27 (2.5)	120 (6.4)
Transgender, *n* (%)	28 (2.6%)	0 (0)

*Body mass index*		
Median (IQR)	28.7 (25.1–32)	24.8 (22.6–27.8)
Data not available, *n* (%)	228 (20.8)	572 (30.5)

*Most common comorbidities, n (%)*		
Hypertension	221 (20.2)	226 (12)
Diabetes mellitus	80 (7.3)	123 (6.5)
Non-AIDS-defining malignancy	43 (3.9)	65 (3.5)
Neurological disease	16 (1.5)	21 (1.1)
Ischemic cardiac disease	9 (0.8)	54 (2.9)

*Method of HIV acquisition, n (%)*		
Heterosexual	947 (86.5)	513 (27.3)
Vertical transmission	38 (3.5)	28 (1.5)
Transfusion	18 (1.6)	15 (0.8)
Homosexual/Bisexual	0 (0)	1119 (59.6)
Intravenous drug use	15 (1.4)	44 (2.3)
Data not available	76 (5.6)	159 (8.5)

*Time since HIV diagnosis (years)*		
Median (IQR)	18 (10.8–23.9)	13.3 (7.2–21)
Data not available, *n* (%)	32 (2.9)	57 (3)

*Prior AIDS defining illness*		
*N* (%)	186 (17)	243 (12.9)

*NadirCD*4^+^*T-cell count (cells/µL)*		
Median (IQR)	238 (132–374)	303 (165–473)
Data not available, *n* (%)	3 (0.3)	7 (0.4)

*HIV treatment status, n (%)*		
Experienced, <6 months	19 (1.7)	60 (3.2)
Experienced, ≥6 months	1059 (96.8)	1796 (95.6)
Naïve	16 (1.5)	22 (1.2)

*Time on ART (years)*		
Median (IQR)	14.8 (7.8–21.9)	10.2 (5.7–18.3)
Data not available, *n* (%)	16 (1.5)	22 (1.2)

*Number of ART regimens received prior to current treatment*		
Median (IQR)	4 (2–7)	3 (2–5.5)
Data not available, *n* (%)	19 (1.7)	30 (1.6)

*Most common ART regimens at index date, n (%)*		
2 NRTIs + 1 INSTI	345 (31.5)	683 (36.4)
2 NRTIs + 1 NNRTI	317 (29)	365 (19.4)
1 INSTI + 1 NRTI	85 (7.8)	236 (12.6)
2 NRTIs + 1 PI	143 (13.1)	199 (10.6)
1 INSTI + 1 NNRTI	31 (2.8)	88 (4.7)

*CD*4^+^*T-cell count at index date (cells/µL), n (%)*		
<200	33 (3)	65 (3.5)
200–349	82 (7.5)	147 (7.8)
350–499	161 (14.7)	271 (14.4)
≥500	818 (74.8)	1395 (74.3)

*CD*4^+^/*CD*8^+^*ratio at index date*		
Median (IQR)	1 (0.7–1.4)	0.9 (0.6–1.2)
Data not available, *n* (%)	68 (6.2)	112 (6)

*HIV-1 viral load (copies/mL), n (%)*		
<50	965 (88.2)	1665 (88.7)
50–199	47 (4.3)	92 (4.9)
≥200	55 (5)	74 (3.9)
Data not available	27 (2.5)	47 (2.5)

IQR, interquartile range; ART, antiretroviral therapy; NRTI, nucleoside/nucleotide reverse transcriptase inhibitor; INSTI, integrase strand transfer inhibitor; NNRTI, non‐nucleoside reverse transcriptase inhibitor; PI, protease inhibitor.

## Data Availability

Data will be available upon request from the corresponding authors.
